# The complete mitochondrial genome of the Tennessee Dace (*Chrosomus tennesseensis*)

**DOI:** 10.1080/23802359.2021.2005480

**Published:** 2021-12-23

**Authors:** Julia E. Wood, Kayla M. Fast, Michael W. Sandel

**Affiliations:** Department of Biological and Environmental Sciences, University of West Alabama, Livingston, AL, USA

**Keywords:** Mitochondrial genome, *Chrosomus tennesseensis*, mitogenome

## Abstract

The Tennessee Dace, *Chrosomus tennesseensis* (Starnes and Jenkins 1988), is a small minnow (Cypriniformes: Leuciscidae) found in the upper Tennessee River watershed and Graves Creek, in the Mobile River watershed. *Chrosomus tennesseensis* occurs sporadically throughout its range and has been listed as vulnerable by the IUCN (NatureServe). Until recently, *C. tennesseensis* had been known only to occur in the upper Tennessee River watershed, however, it has been discovered in headwaters of the Black Warrior River of the Mobile River watershed. We sequenced the mitochondrial genome of *C. tennesseensis* collected in the Mobile River watershed to better understand the colonization of the Mobile River watershed and the interspecific relationships of *Chrosomus*. Furthermore, the availability of the mitochondrial genome will assist in designing specific environmental DNA (eDNA) primers that will allow for less intrusive sampling of threatened and endangered *Chrosomus* species.

The Tennessee Dace, *Chrosomus tennesseensis* (Starnes and Jenkins 1988), had been collected exclusively in the upper Tennessee River watershed until surveys in Graves Creek of the Black Warrior River of the Mobile River watershed uncovered an established population located south of the Tennessee Valley Divide (Wood et al. 2021). Ongoing research is investigating how the species came to colonize Graves Creek in northeast Alabama. Currently, the distribution of *C. tennesseensis* includes north Alabama, eastern Tennessee, southwestern Virginia, western North Carolina, and northwestern Georgia (Etnier and Starnes 1993; Wood et al. 2021). The species prefers first-order tributaries less than two meters wide, leading to a patchy distribution across its known range (Etnier and Starnes 1993). Additionally, several other members of the genus, *C. saylori, C. cumberlandensis,* and *C.* sp. cf. *saylori*, are federally listed as endangered or vulnerable (NatureServe, 2013). The number of endangered or vulnerable species necessitates the design of species-specific eDNA primers to allow for noninvasive sampling methods.

We sequenced the mitochondrial genome of *C. tennesseensis* collected in Graves Creek to better understand the mechanism that led to the colonization of Graves Creek and the relationships between members of the genus *Chrosomus*. Five specimens were collected from Graves Creek (34.045002°N, 86.572004°W). Specimens were anesthetized using MS-222 and whole specimens were preserved in 100% EtOH or 100% acetone, or fin clips were taken and the body was preserved in 10% formalin for museum collections. The specimen used to sequence the mitochondrial genome is stored at the University of Alabama Ichthyological Collection (uaic.as.ua.edu, Manley Worth Pugh, mwpugh@ua.edu) under the voucher number UAIC 49541.01. DNA was extracted using Qiagen DNeasy Blood and Tissue Kits, and the presence of DNA was confirmed using gel electrophoresis and a NanoDrop Spectrophotometer 2000. The specimen with the highest DNA concentration and the brightest band was selected for re-extraction of gill tissue to increase the concentration of mitochondrial DNA.

DNA extracted from gill tissue was prepared for sequencing using the Oxford Nanopore Genomic DNA by Ligation (SQK-LSK110) protocol. We utilized Flongle flow cells for the Oxford Nanopore MinION to sequence both mitochondrial and nuclear DNA. The Flongle flow cell was checked prior to use to determine if there were enough viable pores for sequencing. We used the software MinKNOW (4.2.8) to operate the MinION and sequence the DNA. Passed sequence reads were uploaded to Geneious (11.1.3). Sequence reads with a minimum quality score of 30 were mapped to a *Chrosomus erythrogaster* reference mitochondrial genome sequence (NC031570.1). The consensus mitogenome of *C. tennesseensis* was annotated using the online tool MitoAnnotator (Iwasaki et al. 2013). The generated consensus alignment and 13 additional reference sequences were aligned using MAFFT (Katoh et al. 2019) and further manually checked and aligned using BioEdit (Hall 1999). The *C. tennesseensis* genome has a GC content of 44.68% and a total length of 16,597 bp. Following alignment in MAFFT, the control region was removed from each sequence before phylogenetic analyses were performed since the gene had many gaps that obfuscate homology assessment. The alignment was uploaded to CIPRES and analyzed using RAxML-HPC BlackBox (8.2.12) (Stamatakis 2014) (Figure 1). Nodes with low support were comparable to other studies (Schonhuth et al. 2018). Comparable with other studies that examined a single gene, *C. tennesseensis* was recovered as sister to *C. eos* and *C. erythrogaster* (Strange and Mayden 2009). As expected, *Cyprinus carpio* (Family: Cyprinidae) was recovered as the outgroup and sister to all other included species (Family: Leuciscidae). The availability of the mitochondrial genome will assist in assessing phylogenetic relationships and designing species-specific eDNA primers. Primers designed from our mitochondrial genome could be used as the first step in implementing non-intrusive sampling methods for federally endangered or vulnerable species within the *Chrosomus* genus.

**Figure 1.**
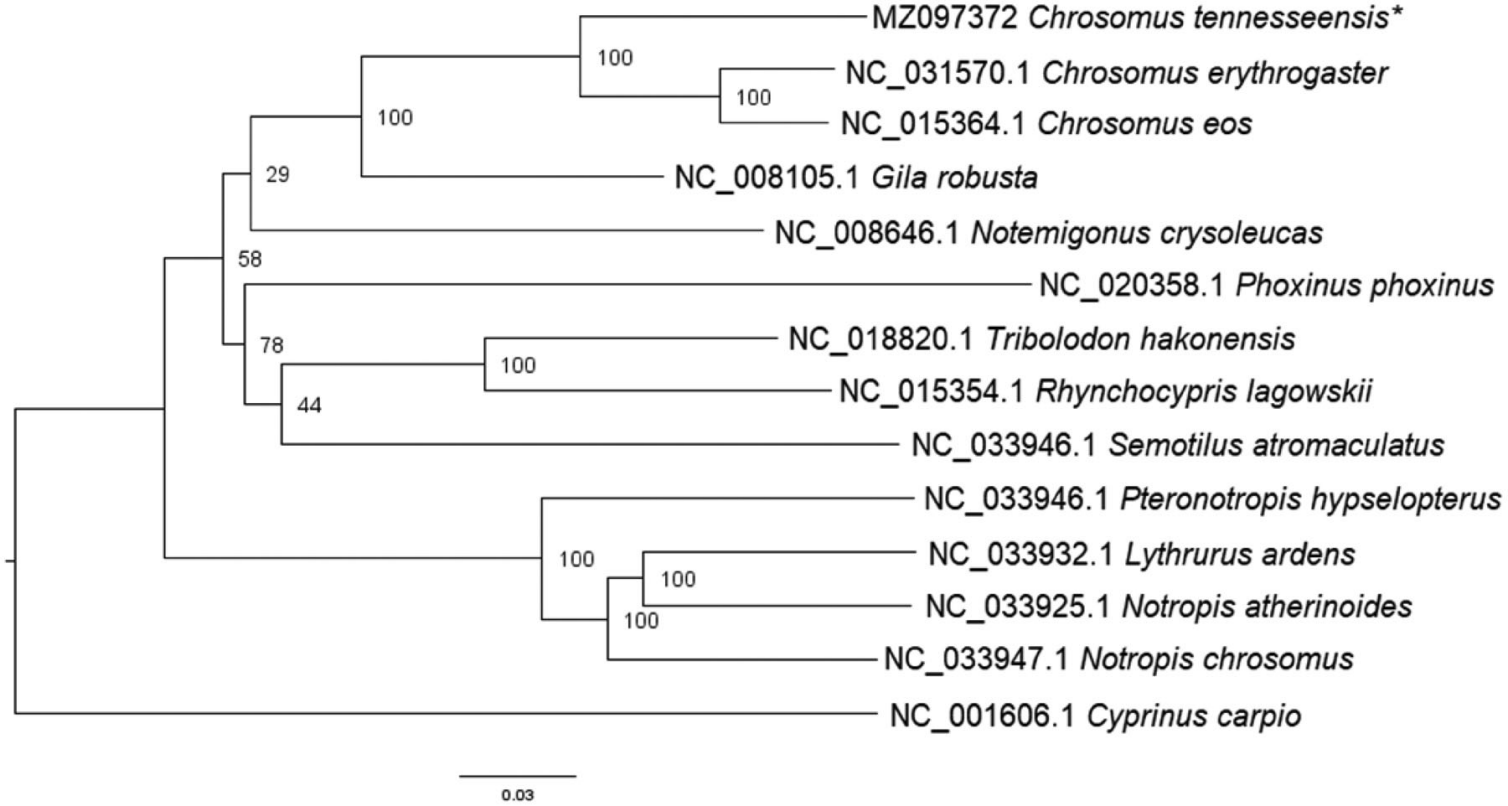
Phylogenetic relationships of several Leuciscid species, including the study species, *Chrosomus tennesseensis,* indicated by an asterisk (*). *Cyprinus carpio* is a member of the family Cyprinidae and is the outgroup in this dataset. The above tree was generated using the maximum likelihood software, RAxML-HPC BlackBox (8.2.12) (Stamatakis 2014).

## Data Availability

The genome sequence data that support the findings of this study are openly available in GenBank of NCBI at (https://www.ncbi.nlm.nih.gov/nuccore/MZ097372.1) under the accession no. MZ097372.1. The associated BioProject, SRA, and Bio-Sample numbers are PRJNA742674, SRX11449843, and SAMN20223869 respectively.

## References

[CIT0001] Etnier DA, Starnes WC. 1993. The fishes of Tennessee. Knoxville (TN): The University of Tennessee Press.

[CIT0002] Hall TA. 1999. BioEdit: a user-friendly biological sequence alignment editor and analysis program for Windows 95/98/NT. Nucleic Acids Symp Ser. 41:95–98.

[CIT0003] Iwasaki W, Fukunaga T, Isagozawa R, Yamada K, Maeda Y, Satoh TP, Sado T, Mabuchi K, Takeshima H, Miya M, et al. 2013. MitoFish and MitoAnnotator: a mitochondrial genome database of fish with an accurate and automatic annotation pipeline. Mol Biol Evol. 30(11):2531–2540. 23955518 10.1093/molbev/mst141 PMC3808866

[CIT0004] Katoh K, Rozewicki J, Yamada KD. 2019. MAFFT online service: multiple sequence alignment, interactive sequence choice and visualization. Brief Bioinform. 20(4):1160–1166. 28968734 10.1093/bib/bbx108 PMC6781576

[CIT0005] NatureServe. 2013. *Chrosomus tennesseensis*. The IUCN Red List of Threatened Species 2013: e.T17065A15362565. [accessed 2021 Jun 6]. 10.2305/IUCN.UK.2013-1.RLTS.T17065A15362565.en.

[CIT0006] Schonhuth S, Vukic J, Sanda R, Yang L, Mayden RL. 2018. Phylogenetic relationships and classification of the Holarctic family Leuciscidae (Cypriniformes: Cyprinoidei). Mol Phylogenet Evol. 127:781–799. 29913311 10.1016/j.ympev.2018.06.026

[CIT0007] Stamatakis A. 2014. RAxML version 8: a tool for phylogenetic analysis and post-analysis of large phylogenies. Bioinformatics. 30(9):1312–1313. 24451623 10.1093/bioinformatics/btu033 PMC3998144

[CIT0008] Strange RM, Mayden RL. 2009. Phylogenetic relationships and a revised taxonomy for North American cyprinids currently assigned to Phoxinus (Actinopterygii: Cyprinidae). Copeia. 2009(3):494–501.

[CIT0009] Wood JE, Pugh MW, Harris PM, McGregor SW, Sandel MW. 2021. Range extension of blackfin darter and Tennessee Dace, and first collection of western Blacknose Dace from Locust Fork of the Black Warrior River in 80 years. Southeastern Nat. 20(1):N30–N36.

